# Detection of swine transmissible gastroenteritis coronavirus using loop-mediated isothermal amplification

**DOI:** 10.1186/1743-422X-7-206

**Published:** 2010-08-29

**Authors:** Qin Chen, Jian Li, Xue-En Fang, Wei Xiong

**Affiliations:** 1 School of Life Science, Shanghai University, Shanghai 200444, China; 2 Shanghai Entry-Exit Inspection and Quarantine Bureau, Shanghai 200135, China

## Abstract

A conserved nucleic acid fragment of the nucleocapsid gene of Swine Transmissible Gastroenteritis Coronavirus (TGEV) was chosen as the target, six special primers were designed successfully. Loop-mediated isothermal amplification (LAMP) was developed to detect the TGEV by incubation at 60°C for 1 h and the product specificity was confirmed by *Hph*I digestion. Standard curves with high accuracy for TGEV quantization was constructed by adding 1 × SYBR greenI in the LAMP reaction. The assay established in this study was found to detect only the TGEV and no cross-reaction with other viruses, demonstrating its high specificity. By using serial sample dilutions as templates, the detection limit of LAMP was about 10 pg RNA, 10 times more sensitive than that of PCR and could be comparable to the nest-PCR.

## Background

Swine Transmissible Gastroenteritis Coronavirus (TGEV), as a member of the coronaviridae, is a kind of single-stranded RNA virus, which produces villous atrophy and enteritis, leading to the serious financial loss to the whole pig industry. The traditional detection methods, including virus isolation, virus immunodiagnostic assays and PCR tests have the shortcomings, such as precise instruments requirement, elaborate result analysis demand, high cost, long detection time and so forth, which prevent these methods from being widely used[1-4]. Loop-mediated isothermal amplification (LAMP) is a novel nucleic acid amplification method, which amplifies DNA/RNA with high specificity, sensitivity and rapidity under isothermal condition [5]. It has already found wide application in RNA virus detection, such as Foot-and-mouth Disease Virus[6], Swine Vesicular Disease Virus[7], Taura Syndrome Virus [8], Severe Acute Respiratory Syndrome Coronavirus and H5N1 Avian Influenza Virus[9,10]. In this study, LAMP method was applied in developing qualitative and quantitative detection system of TGEV, while its specificity and sensitivity were assessed.

## Methods

### Samples

Swine Transmissible Gastroenteritis Coronavirus (TGEV, strain H), Porcine Reproductive and Respiratory Syndrome Virus (PRRSV), Pesudorabies (PRV), Porcine Parvovirus (PPV) derived from their passages in cell culture were provided by Shanghai Entry-Exit Inspection and Quarantine Bureau (SHCIQ); nucleic acids of Foot-and-mouth Disease Virus (FMDV) and Classical Swine Fever Virus (CSFV) were obtained from Chinese Academy of Inspection and Quarantine (CAIQ).

### RNA/DNA extraction

Total genomic RNA was extracted using Trizol Kit (Invitrogen, USA). DNA was extracted by DNA Blood Mini Kit (Qiagen, Germany). After elution in 20 μL Nuclease-free Water, RNA/DNA samples were stored at -70°C before use. The original concentration of RNA/DNA sample was about 50 ng/μL.

### Target region and LAMP primers designing

Complete genome sequences of fifteen different TGEV strains/isolates and nine other similar viruses were obtained from GenBank, and the homology was analyzed using the Vector NTI. The conserved fragment with high homology was chosen as the target region which and used to design the TGEV LAMP primers by the Primer Explorer V3 software http://primerexplorer.jp/e/.

### The construction of standard control

The target RNA of TGEV was first reverse transcripted using Superscript™ II (Invitrogen, USA) and then amplified by Pfu DNA polymerase using forward primer: GGAAGAGAACTGCAGGTAA and reverse primer: CCATCTTCCTTTGAAGTCCA. The amplified product was purified from agarose gels and then cloned into *E. coli *JM109 using the pMD18-T vector. The target plasmid with the original concentration of 8.67 × 10^8 ^Copies/μL was extracted by the Plasmid Mini Preparation Kit and identified by the 260 nm absorption spectroscopy, which was then used as the standard for the quantitative analysis.

### LAMP

The LAMP reaction was carried out in a volume of 25 μL containing 1 × ThermoPol Buffer (New England Biolabs, USA), 8.0 mM MgSO4, 0.8 M Betaine (Sigma, Germany), 1.2 mM dNTPs, 0.2 μM each of Outer primers, 1.6 μM each of Inner primers and 0.4 μM each of Loop primers, 5 U AMV Reverse Transcriptase, 8 U of *Bst *Polymerase (Large Fragment; New England Biolabs, USA) with 2 μL total RNA as template. The amplification was performed at 60°C in a laboratory water bath (Kangle, China, 25°C~99°C, with temperature accuracy of ±0.3°C) for 1 h.

The amplified products were digested with *Hph*I to confirm its specificity.

The result of TGEV-LAMP was analyzed by agarose gel electrophoresis and fluorescence by adding 1 × SYBR greenI in the LAMP reaction.

### LAMP evaluation

The specificity of TGEV-LAMP was examined by the use of RNA (or DNA) extracted from five other pig disease viruses. The sensitivity of TGEV-LAMP was evaluated by comparing with PCR[4] and Nest-PCR[11], using 10-serial TGEV RNA dilutions (10^-1 ^to 10^-7^) as templates.

## Results

### LAMP primers

**Figure 1:**
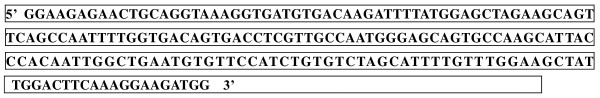
**The conserved fragment of the nucleocapsid gene from TGEV**.

LAMP primers were designed using the Primer Explorer V3 software based on a conserved fragment of the nucleocapsid gene (Fig. 1). The primers including Outer Primers (F3 and B3), Inner primers (FIP and BIP) and Loop primer (LF and LB) were shown in Table 1.

**Table 1:** TGEV LAMP primers

Primer name	Type	Length/bp	Sequence(5'to 3')
TGEV-F3	Forward Outer	19	GGAAGAGAACTGCAGGTAA
TGEV-B3	Reverse Outer	20	CCATCTTCCTTTGAAGTCCA
TGEV-FIP	Forward Inner	45	CGAGGTCACTGTCACCAAAATT
			TGATGTGACAAGATTTTATGGAG
TGEV-BIP	Reverse Inner	42	GGAGCAGTGCCAAGCATTAC
			AAAATGCTAGACACAGATGGAA
TGEV-LF	Forward Loop	17	GGCTGAACTGCTTCTAG
TGEV-LB	Reverse Loop	19	CCACAATTGGCTGAATGTG

### Detection of TGEV by LAMP

**Figure 2:**
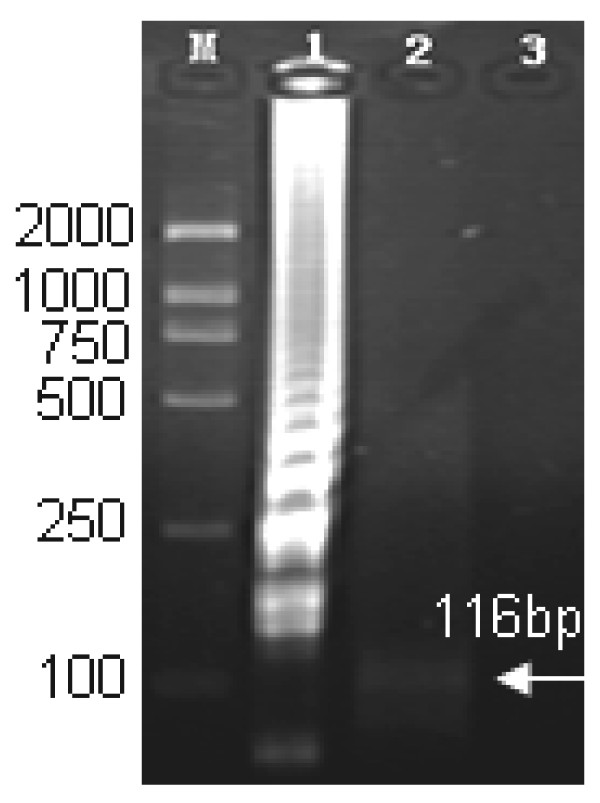
**Analysis of TGEV by LAMP with agarose gel electrophoresis**. 1, LAMP products of TGEV; 2, LAMP products digested with *Hph *I; 3, Negative control.

TGEV deprived from the cell culture was first qualitatively analyzed by LAMP. Amplification products were analyzed by agarose gel electrophoresis. As shown in Fig.2 (Lane 1), amplification could be carried out at 60°C and showed a ladder-like pattern on the gel while the negative control gave no bands (Lane 3). The specificity of the LMAP product was confirmed by *Hph*I digestion. Predictable product of the 116-bp motif was resolved on the gel as theoretical expected (Fig.2, lane 2).

**Figure 3:**
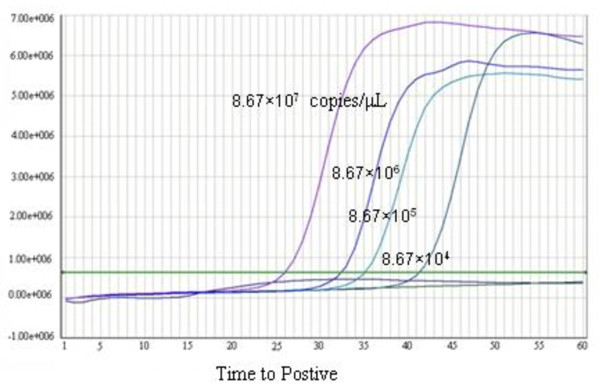
**Dynamic curves of the different TGEV standards**.

**Figure 4:**
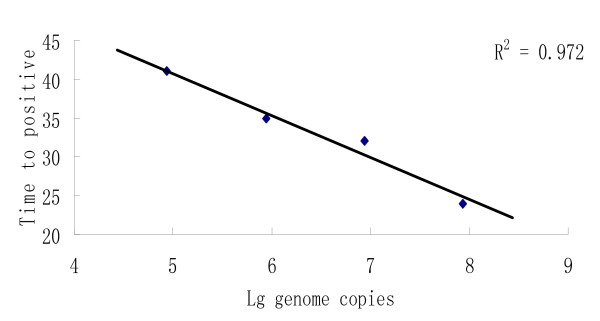
**Standard curves of real-time fluorescence TGEV-LAMP**.

Standard control was used to develop real-time fluorescence LAMP for quantitatively analyzing TGEV. Dynamic curves for TGEV quantification was generated by serially diluting the standard control from 8.67 × 10^7 ^to 8.67 × 10^4 ^copies/μL(Fig. 3). The log linear regression plot (standard curves) was obtained by plotting the time-to-positive (TTP) values against genome copies. The correlation coefficients were 0.972 (Fig. 4)

### Evaluation of LAMP

**Figure 5:**
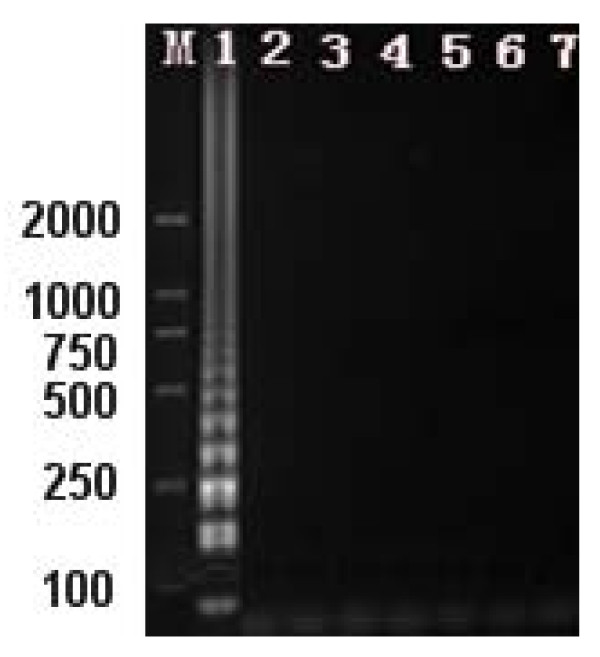
**Specificity of LAMP for TGEV detection**. 1-6, TGEV, PRRSV, PRV, CSFV, PPV and FMDV respectively; 7, Negative Control.

**Figure 6:**
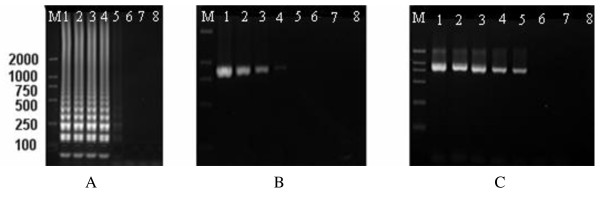
**Sensitivity of LAMP for TGEV detection**. A, LAMP; B, PCR; C, Nest-PCR; 1-7, TGEV RNA sample at 10^-1^, 10^-2^...10^-7 ^dilutions respectively; 8. Negative Control.

Five other pig viruses were used to confirm the specificity of the LAMP for TGEV detection. The results showed that only TGEV detected gave amplification products while no amplification available to other viruses (Fig.5). The sensitivity of LAMP was demonstrated by comparing with PCR tests using serial dilutions (10^-1 ^to 10^-7^) of TGEV RNA samples as template. As shown in Fig.6, LAMP and nest-PCR were able to detect 10^-5 ^dilution (about 10 pg RNA), whereas PCR could only amplify the 10^-4 ^dilution. Therefore, the sensitivity of TGEV-LAMP could be comparable to nest-PCR, 10-fold higher than PCR.

## Discussion

It is very important to find out a conserved nucleic acid fragment for designing specific LAMP primers and developing efficient, accurate LAMP assay [12,13]. In this study, the nucleic acid sequence homology of 15 TGEV strains/isolates and 9 other similar viruses available from GenBank were analyzed by Vector NTI software. The most conserved fragment of 187 bp was found in the nucleocapsid protein gene which showed highly homology among different TGEV strains/isolates (more than 97%) and low homology among other similar viruses (less than 52.5%). The TGEV LAMP primers targeting the conserved sequence were designed successfully by the Primer designer V3 software.

The target region was amplified successfully by the LAMP with a characteristic ladder-like pattern of bands from 187 bp on the gel. This is because the final products of LAMP are a mixture of stem-loop DNAs with various stem lengths and cauliflower-like structures with multiple loops formed by annealing between alternately inverted repeats of the target sequence in the same strand [5,14]. After digestion with *Hph*I, 116-bp motif was resolved on the gel as expected, demonstrating the specific structure of amplification products, which could also be validated by nucleic acid sequencing. As a kind of nucleic acid amplification method, LAMP could not only qualitatively detect the TGEV, but also quantitatively analyze the virus. In this study, real time fluorescence LAMP for quantitatively detection of TGEV was established by adding 1 × SYBR greenIin the LAMP reaction. Three standard curves established by TGEV standards displayed the good correlation between the TTP and virus copies, implicating the great potential in quanlitatively detecting TGEV.

Five other viruses were used in this study to confirm the specificity of LAMP. The results showed no amplification in all viruses tested, which makes the LAMP more accurate and reliable for TGEV detection. The high specificity of LAMP is most probably attributable to recognition of the target sequence by six independent sequences in the initial stage and by four independent sequences during the second reaction stage. The sensitivity of LAMP was evaluated using various TGEV-RNA dilutions as templates. The assay exhibited almost equivalent sensitivity to the nest-PCR and 10-fold higher than the PCR, indicating that the LAMP is a more powerful diagnosis tool to detect TGEV in lower copy conditions [11]. In addition, the TGEV-LAMP developed has advantages in its rapid detection and simple operation. The only equipment required for the reaction is a water bath or heat block. The assay developed is a faster detection method for the TGEV detection, only taking about 1 h, which means the whole diagnosis including RNA extraction, amplification and product detection could be completed within one and a half hour after receiving of the samples. It is anticipated that with the advantages of specificity, sensitivity, reliability, rapidity and easy manipulation, LAMP will turn out be a powerful molecular tool for the TGEV detection in practice.

## Conclusions

In conclusion, this study demonstrates that the LAMP method established could detect only the TGEV and no cross-reaction with other viruses, the detection limit was about 10 pg RNA, which was 10 times more sensitive than that of PCR and could be comparable to the nest-PCR.

## Competing interests

The authors declare that they have no competing interests.

## Authors' contributions

Conceived and designed the experiments: QC, JL, X-EF. Performed the experiments: X-EF, JL. Analyzed the data: JL, X-EF, WX. Wrote the paper: QC, X-EF. All authors read and approved the final manuscript.
